# Intraoperative End-Tidal Carbon Dioxide Concentrations: What Is the Target?

**DOI:** 10.1155/2011/271539

**Published:** 2011-10-25

**Authors:** Megan Way, Gary E. Hill

**Affiliations:** Department of Anesthesiology and Pain Management, The University of Texas Southwestern Medical Center at Dallas, Dallas, TX 75390-9068, USA

## Abstract

Recent publications suggest that target end-tidal carbon dioxide concentrations should be higher than values currently considered as acceptable. This paper presents evidence that end-tidal carbon dioxide values higher than concentrations that are currently targeted result in improved patient outcomes and are associated with a reduced incidence of postoperative complications.

Since the reports of benefits of permissive hypercapnia in the critical care literature [1], the current practice of maintaining mild hypocapnia (i.e., end-tidal carbon dioxide [ETCO_2_] values between 30 and 35 mmHg) is being questioned. This paper will discuss the concerns with mild hyperventilation and hypocapnia and will propose maintenance of higher ETCO_2_ values based on recent literature. Also, the importance of the relationship between cardiac output and ETCO_2_, in particular within the context of low cardiac output states, including weaning from cardiopulmonary bypass (CPB), will be discussed.

In an effort to determine the origin for the current practice of mild hypocapnia, a literature search with the key words intraoperative, general anesthesia, hypocapnia, hypocarbia, or hyperventilation was performed. However, it failed to find peer-reviewed articles describing well-defined benefits of mild hypocapnia during general anesthesia. The use of mild hypocapnia may have been introduced because of the concerns of hypercapnia-related hypertension and tachycardia that may increase myocardial O_2_ demand, which may lead to myocardial ischemia and infarction [2]. Other proposed benefits of intraoperative hypocapnia include reduction in the need for muscle relaxants as well as reduced hypnotic requirements. However, there is no good evidence for such claims. Despite the lack of peer-reviewed data, mild hypocapnia remains accepted in clinical practice. 

Classic scientific publications by Seymour Kety and Carl Schmidt [3, 4] in the 1940s demonstrated the well-known curvilinear increase of cerebral blood flow (CBF) with increasing arterial carbon dioxide (PaCO_2_) concentrations, secondary to reduced cerebral vascular resistance, as well as increases in cardiac output (CO) and systemic arterial blood pressure (ABP) caused by hypercapnia. Hypocapnia, on the other hand, caused the opposite effects: reduction of CBF and an increase in cerebral oxygen (O_2_) consumption, secondary to cerebral vasoconstriction and reduced CO. These foundational publications were followed closely with another classic paper by Cournand et al. [5] demonstrating that a reduction of CO occurred following the application of positive pressure ventilation. Thus, it has been well established for over 60 years that positive pressure ventilation with ensuing hypocarbia has the undesirable consequences of the reduction of CO, mean arterial blood pressure and CBF. 

The detrimental effects of hyperventilation and hypocapnia are many. Initiation of hyperventilation immediately after induction of anesthesia, which causes vasodilatation and relative hypovolemia, may cause significant reduction in venous return, underfilling of the right heart, and subsequent reduction of CO and ABP [6]. In addition, hypocapnia may cause prolongation of the QT interval and cardiac arrhythmias [7]. Other effects of hypocapnia include increases in lung microvascular permeability, decreased lung compliance (due to bronchoconstriction), increased intrapulmonary shunt fraction caused by inhibition of hypoxic pulmonary vasoconstriction, and leftward shift of the oxyhemoglobin dissociation curve, as well as worsened outcome after acute head injury and cardiopulmonary resuscitation [8]. 

Otherwise healthy patients subjected to intraoperative hypocapnia (PaCO_2_ = 24 mmHg) during routine general anesthesia demonstrate cognitive dysfunction for several days postoperatively [9]. A control group maintained with PaCO2 levels greater than 24 mmHg did not demonstrate any signs of cognitive dysfunction. Hovorka [10] reported in female patients that the hypercapnic group (PaCO_2_ = 54 mmHg) was found to have better cognitive function scores when compared to both a normocapnic group (PaCO_2_ = 40 mmHg) and two hypocapnic groups (PaCO_2_ = 28 in patients older than 60 yrs and 22 mmHg in patients under 46 yrs of age). Also, the normocapnic group had better cognitive function scores than both hypocapnic groups. Even short duration hypocapnia during cesarean section has negative effects on the fetus, including decreased fetal PaO_2_ levels, increased base deficit, and lower Apgar scores [8]. 

Because hypocarbia is known to reduce CBF, Rosner et al. [11] have focused on cerebral perfusion pressure management of traumatic brain injury (TBI) rather than standard hyperventilation management and have reported favorable outcomes. Despite current guidelines recommending against it [12], deliberate hyperventilation continues to be routinely used for TBI. In fact, over one-third neurosurgeons and half emergency physicians still utilize hyperventilation for ICP control after TBI. 

In contrast to the detrimental effects of hypocapnia, mild hypercapnia has number of benefits. Mild respiratory acidosis demonstrates a protective effect against organ injury, and targeting a normal (or lower than normal) PaCO_2_ may be injurious [13]. Mild hypercapnia can improve tissue oxygenation through improved tissue perfusion resulting from increased CO and vasodilatation as well as increased O_2_ off-loading from the shift of the oxyhemoglobin dissociation curve to the right. Akça et al. [14] reported increased subcutaneous tissue O_2_ tension (PsqO_2_) measured in the upper arm with mild hypercapnia. Their data demonstrated a linear increase of PsqO_2_ with increasing ETCO_2_, with the greatest increase of PsqO_2_ at an ETCO_2_ of 60 mmHg. Since PsqO_2_ is significantly correlated with the risk of surgical infections, with higher PsqO_2_ resulting in lower infection rates, this implies an important benefit of mild hypercapnia in reducing postoperative infection rates. Mild hypercapnia similarly improves PsqO_2_ in the morbidly obese [15]. Mild hypercapnia (ETCO_2_ 50 mmHg) along with increased inhaled O_2_ concentration (80%) resulted in increased subcutaneous and colonic tissue O_2_ tensions in patients during colon resection [16], implying such therapy may prove to be beneficial in reducing infection rates and a more rapid return to normal bowel function. 

Several investigators have reported improved lung function (e.g., reduced intrapulmonary shunt) in patients with respiratory distress syndrome using a protective ventilatory strategy (i.e., permissive hypercapnia, PaCO_2_ of ±50 mmHg) when compared to control groups managed in a traditional manner (i.e., PaCO_2_ of ±35 mmHg) [1]. Respiratory acidosis (hypercapnia) attenuates or dampens inflammation caused by established bacterial pneumonia [17], ischemia-reperfusion, and endotoxin-induced lung injury. 

It has been well established that large tidal volume ventilation causes a stretch type of injury (volutrauma) resulting in lung inflammation characterized by proinflammatory cytokine release, airway edema, and increased extravascular lung water. Lung injury, measured by increased lung weight and hemorrhage scores, is also reduced by decreased respiratory frequency (ventilatory rate) in animals [18]. While the relationship between proinflammatory cytokine release and ventilator-induced lung injury remains to be clearly defined [19], there seems to be a general consensus of opinion that proinflammatory cytokines may be involved in positive pressure ventilation-induced lung injury. 

Sakata et al. [20] have utilized hypercapnia (achieved by rebreathing exhaled CO2) in conjunction with hyperventilation to shorten recovery times following sevoflurane and desflurane anesthesia. 

While mild hypercapnia has well-defined clinical benefits, it must be avoided in certain clinical scenarios. Clearly, mild hypercapnia would further exacerbate elevated intracranial pressure. In addition, hypercapnia may lead to significant diaphragm muscle dysfunction/fatigue [21]. Furthermore, hypercapnic respiratory acidosis may cause increased difficulty in the reversal of nondepolarizing neuromuscular blocking agents by neostigmine [22]. Interestingly, an author of one of the original reports on the negative consequences of hypercapnia [23] published a creative and unique paper investigating the mechanism of brain injury found after mountain climbing to extreme altitudes [24]. The surprising results demonstrated that it was not hypoxemia that caused brain injury but hypocapnic-induced reduction of CBF as the primary mechanism of CNS injury after climb, dramatizing the serious negative consequences of hypocapnia on normal brain function. 

Falk et al. [25] were the first to report that ETCO_2_ could be used to predict outcomes following cardiopulmonary resuscitation (CPR). Their data demonstrated rapidly increasing (within 30 seconds following initiation of CPR) ETCO_2_ was predictive of survival, while continuing low levels of ETCO_2_ predicted death. Similarly, Levine et al. [26] found that ETCO_2_ levels of 32.8 + 7.4 mmHg predicted survival while low ETCO_2_ levels (4.4 + 2.9 mmHg) predicted death following 20 min of CPR. Levine et al. [26] also found that an ETCO_2_ level of 10 mmHg or less was 100% accurate when predicting death following 20 min of CPR. Other clinical scenarios characterized by low cardiac output (hemorrhage or weaning from cardiopulmonary bypass (CPB)) have demonstrated a similar direct correlation between cardiac output and ETCO_2_. Dubin et al. [27] demonstrated a direct linear correlation between CO and ETCO_2_ during progressive hemorrhage in an anesthetized dog model. In humans with normal preoperative lung function, Maslow et al. [28] reported a significant direct correlation between CO and ETCO_2_ during weaning from CPB. Their data demonstrated that when ETCO_2_ was greater than 30 mmHg, CO was greater than 4.0 L/min, and when ETCO_2_ was greater than 34 mmHg, CO was greater than 5 L/min. 

Similar findings were reported by Feng and singh. [29] who found that separation from CPB was successful in patients whose ETCO_2_ was consistently near or greater than 30 mmHg. 

In conclusion, the dogma of maintaining ETCO_2_ values between 30 and 35 mmHg is without scientific merit and needs to be revisited. In fact, hypocapnia, and the hyperventilation required to achieve it, is clearly not benign. On the other hand, mild hypercapnia (ETCO_2_ values around 40 mmHg or higher, but with the caveats as previously described) is beneficial and should come to be accepted as the standard of care. 
